# Cost-utility analysis of PCSK9 inhibitors for hypercholesterolemia: a Chinese healthcare perspective

**DOI:** 10.3389/fphar.2025.1708701

**Published:** 2025-11-19

**Authors:** Dandan Hu, Xu Deng, Mengwen Feng, Xinyue Zhang, Qingfeng He, Tao Wang, Zhen Feng

**Affiliations:** 1 Department of Pharmacy, The Affiliated Hospital of Xuzhou Medical University, Xuzhou, Jiangsu, China; 2 Department of Pharmacy, Southeast University Affiliated Xuzhou Central Hospital, Xuzhou, Jiangsu, China; 3 Department of Cardiology, The Affiliated Hospital of Xuzhou Medical University, Xuzhou, Jiangsu, China; 4 School of Public Health, Fudan University, Shanghai, China; 5 Department of Clinical Pharmacy and Pharmacy Administration, School of Pharmacy, Fudan University, Shanghai, China

**Keywords:** PCSK9 inhibitors, hypercholesterolemia, cost-utility, pharmacoeconomics, Markov model, China

## Abstract

**Objective:**

To evaluate the cost-utility of different dosing regimens of PCSK9 inhibitors, added to statin therapy, in patients with hypercholesterolemia or at high cardiovascular risk in China.

**Methods:**

A Markov cohort multistate-transition model was developed from the perspective of the Chinese healthcare system, with a 1-year cycle and lifetime horizon. Treatment effects were derived from a network meta-analysis. Costs, utilities, and mortality data were obtained from published literature and national databases. Both costs and outcomes were discounted at a rate of 5% annually. The primary outcome was the incremental cost-utility ratio (ICUR). The willingness-to-pay (WTP) threshold was set at 1–3 times China’s 2024 *per capita* gross domestic product. One-way, probabilistic, and scenario sensitivity analyses were performed to test model robustness.

**Results:**

In the base-case analysis, evolocumab 140 mg every 2 weeks (Q2W) was the most cost-effective option, dominating all other active regimens. Compared with statin therapy alone, it generated incremental costs of $11,109.27 and a quality-adjusted life year (QALY) gain of 0.42. The resulting ICUR was $26,217.47 per QALY, which is below the WTP threshold of $39,875.0 per QALY. Although less favorable than evolocumab, alirocumab 75 mg Q2W and tafolecimab 150 mg Q2W were also cost-effective versus statins alone, with ICURs of $34,279.73 and $34,002.10 per QALY, respectively. All other regimens were dominated, and inclisiran showed the least favorable cost-utility profile (ICUR $113,800.14 per QALY). Sensitivity analyses identified the discount rate as the key driver of uncertainty, with ICURs for evolocumab 140 mg Q2W versus statins alone ranging from $15,903.46 to $34,573.62 per QALY. Probabilistic sensitivity analysis showed a 98.9% probability of evolocumab 140 mg Q2W being cost-effective versus statins alone.

**Conclusion:**

At the current negotiated price, evolocumab 140 mg Q2W is the most cost-effective PCSK9 inhibitor regimen for Chinese patients with hypercholesterolemia or at high cardiovascular risk when added to statin therapy. Alirocumab 75 mg Q2W and tafolecimab 150 mg Q2W also represent cost-effective alternatives. These findings provide important evidence to support clinical decision-making and optimize resource allocation in China.

## 1 Introduction

Cardiovascular disease (CVD) remains the leading chronic non-communicable disease worldwide. In China, atherosclerotic cardiovascular diseases (ASCVD), including ischemic heart disease and ischemic stroke, are the primary causes of death in both urban and rural populations, accounting for over 40% of total mortality (National Center for Cardiovascular Diseases, 2022). Despite advances in prevention and treatment, the burden of ASCVD in China continues to rise (Zhao et al., 2019), underscoring the urgent need for more effective interventions.

Extensive epidemiological, genetic, and clinical evidence has firmly established low-density lipoprotein cholesterol (LDL-C) as a causal risk factor for ASCVD (Ference et al., 2017). However, while the prevalence of dyslipidemia among Chinese adults has increased substantially (Song et al., 2019), awareness, treatment, and control rates remain low (Zhang et al., 2018). This mismatch between disease burden and lipid control highlights the need for more effective lipid-lowering strategies to improve cardiovascular outcomes.

Statins remain the cornerstone of LDL-C-lowering therapy, but many Chinese patients fail to achieve LDL-C targets even with high-intensity regimens. This reflects both lower statin tolerability and a higher incidence of adverse effects among Chinese patients (Gitt et al., 2016; HPS2-THRIVE Collaborative Group, 2013). Moreover, doubling the statin dose only yields an additional ∼6% LDL-C reduction while increasing the risk of adverse effects, including liver dysfunction, myopathy, and new-onset diabetes (Stroes et al., 2015; Maki et al., 2014). As a result, Chinese guidelines recommend standard or moderate-intensity rather than high-intensity statin therapy.

The advent of proprotein convertase subtilisin/kexin type 9 (PCSK9) inhibitors has transformed lipid management. By inhibiting PCSK9-mediated degradation of LDL receptors (LDLR), these therapies increase LDL-C clearance, lowering LDL-C levels by 50%–70% and reducing the risk of major adverse cardiovascular events (MACE) when added to statins (Robinson et al., 2015; Ference et al., 2017). Approved agents include monoclonal antibodies (evolocumab and alirocumab) and small interfering RNA (siRNA) agents such as inclisiran.

Landmark trials have confirmed the efficacy of PCSK9 inhibitors in both LDL-C reduction and cardiovascular risk mitigation (Sabatine et al., 2017; Schwartz et al., 2018). Evolocumab, the first approved agent, lowers LDL-C by 59% on top of statins, reduces cardiovascular events by 20%, and achieves a 66% LDL-C reduction in Asian populations (Sabatine et al., 2017; Keech et al., 2021). Over a long-term follow-up, alirocumab consistently reduced LDL-C by nearly 50% and significantly lowered MACE risk (Goodman et al., 2023). Tafolecimab, China’s first domestically developed PCSK9 monoclonal antibody, offers flexible dosing every 2, 4, or 6 weeks to improve treatment adherence (Liu et al., 2025). The CREDIT phase III trials showed that tafolecimab plus statins reduced LDL-C by up to 70% and lipoprotein(a) [Lp(a)] by 50% (Huo et al., 2023; Chai et al., 2023; Qi et al., 2023). Inclisiran, a siRNA therapy, achieves comparable LDL-C reductions to monoclonal antibodies with a single injection maintaining efficacy for up to 6 months (Fitzgerald et al., 2017), offering significant adherence advantages. However, it has not yet been included in the 2025 National Reimbursement Drug List (NRDL), limiting patient access in China.

Despite their clinical benefits, the high costs of PCSK9 inhibitors raise concerns about economic sustainability, especially in resource-limited settings. As new PCSK9 inhibitors and dosing regimens emerge, clinicians face increasing complexity in balancing efficacy, safety, adherence, and cost-effectiveness when selecting optimal therapies. To date, no study has comprehensively compared the cost-utility of different PCSK9 inhibitor regimens within the Chinese healthcare system.

To address these challenges, we developed a Markov cohort state-transition model to compare the cost-utility of various PCSK9 inhibitor regimens combined with statins for Chinese patients with hypercholesterolemia or at high cardiovascular risk. Our aim was to provide robust evidence to inform clinical decision-making, optimize healthcare resource allocation, and support policy development in China.

## 2 Methods

This study was presented per the Consolidated Health Economic Evaluation Reporting Standards 2022 (CHEERS 2022) checklist (Supplementary Table S1) (Husereau et al., 2022).

### 2.1 Model structure

A Markov cohort state-transition model was established to compare the cost-effectiveness of statins in combination with PCSK9 inhibitors versus statins alone for patients with hypercholesterolemia. The model structure was adapted from previously published economic evaluation frameworks of lipid-lowering therapies (Wan et al., 2023; Xie et al., 2023; Wan et al., 2025), and was constructed in TreeAge Pro Healthcare Version 2022 (TreeAge Software, LLC., Williamstown, MA, United States).

**FIGURE 1:**
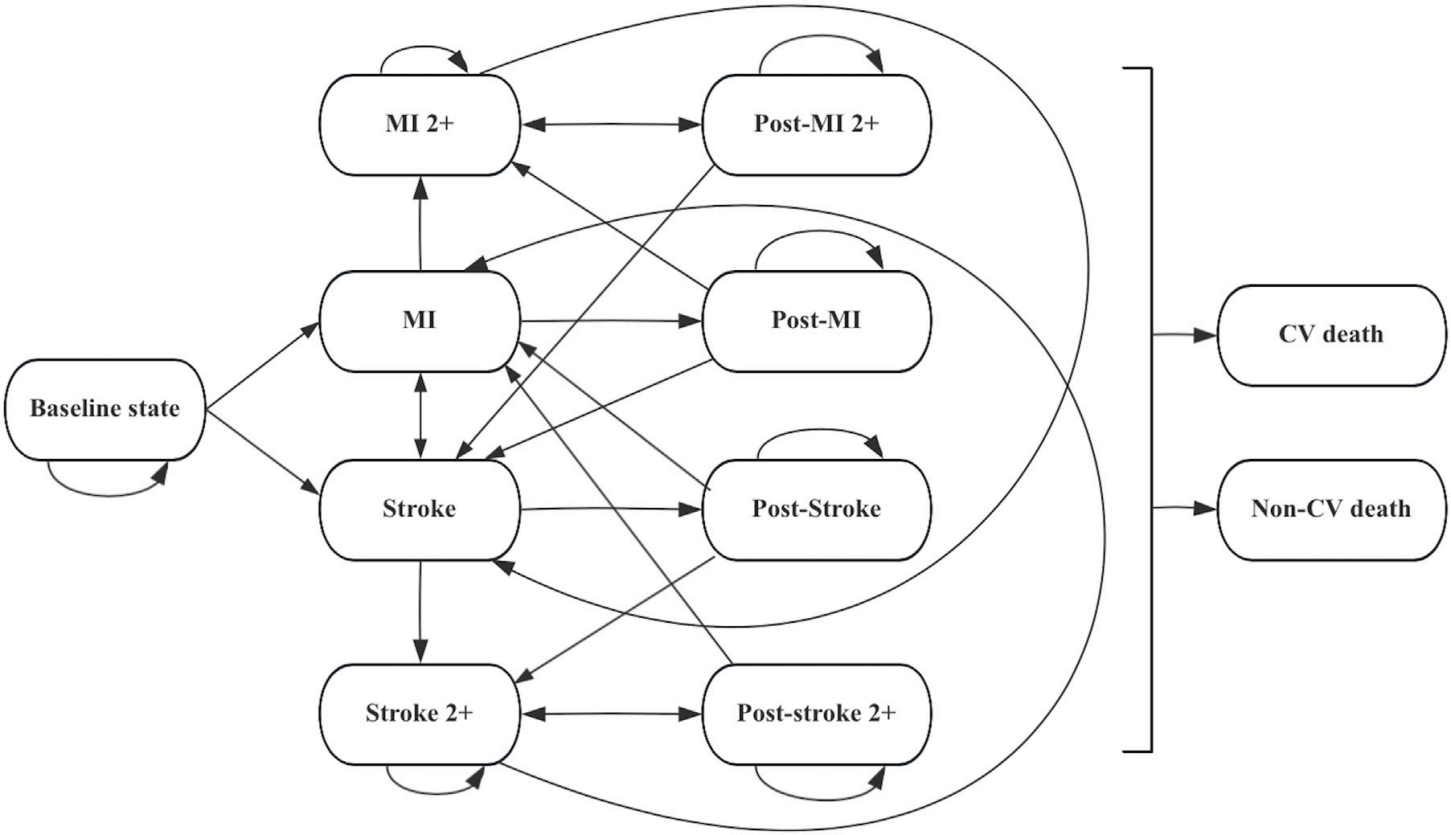
Markov model diagram for hypercholesterolemia patients. Abbreviations: CV cardiovascular, MI myocardial infarction.

The model consisted of 11 mutually exclusive health states to capture the long-term occurrence, progression, and outcomes of cardiovascular events (CVEs). Patients entered the model in the baseline state of being alive without CVD and could either remain in this state or experience a first myocardial infarction (MI) or ischemic stroke (IS). After the first event, patients transitioned into the corresponding post-event state (post-MI or post-IS) and could experience recurrent CVEs, including second or subsequent MI (MI 2+) or IS (IS 2+). In each health state, patients were at risk of transitioning to death, which was classified as either cardiovascular (CV)-related death or non-CV-related death, both considered irreversible absorbing states. See Figure 1 for detailed information.

The model applied a 1-year cycle length and a 30-year lifetime horizon to approximate lifetime cardiovascular risk. It was assumed that only one CV event could occur per cycle, but patients could experience multiple recurrent events across the entire time horizon.

### 2.2 Patient population

This study population was defined based on a network meta-analysis that evaluated the efficacy and safety of PCSK9 inhibitors in patients with hypercholesterolemia or at high cardiovascular risk whose LDL-C levels remained inadequately controlled despite standard lipid-lowering therapy. This network meta-analysis included 26 randomized controlled trials with a total of 16,510 participants. Across trials, the mean age varied from 49.0 to 66.1 years (mean age: 60.6 years), the proportion of female participants ranged between 17.6% and 57.3%, and the mean baseline LDL-C level was 3.13 mmol/L (Liu et al., 2024) (Supplementary Table S2).

All patients were assumed to receive high-intensity statin therapy (atorvastatin 40–80 mg/day or rosuvastatin 20 mg/day, administered orally once daily) as background lipid-lowering treatment. The intervention group received statins in combination with one of the PCSK9 inhibitor regimens, comprising 11 dosing strategies across four agents: alirocumab (75 mg every 2 weeks, 150 mg every 2 weeks, 300 mg every month), evolocumab (140 mg every 2 weeks, 420 mg every month), tafolecimab (150 mg every 2 weeks, 450 mg every 4 weeks, 600 mg every 6 weeks), and inclisiran (300 mg on day 1, day 90, and every 6 months thereafter). The control group received statin therapy alone without the addition of a PCSK9 inhibitor.

### 2.3 Clinical event probabilities and intervention effects

Baseline CV event rates were derived from the statin arm of the Cholesterol Treatment Trialists’ Collaboration (CTTC) meta-analysis (Cholesterol Treatment Trialists' Collaboration et al., 2010). To reflect our target population, we adjusted these rates for age and LDL-C levels using the following equation (Equation 1) (Danese et al., 2021):

$$r_{a}=r_{0}\times {{HR}_{age}^{\left(\Delta age\right)}\times RR}^{\Delta LDLc}$$ (1)

where $r_{a}$ is the adjusted baseline event rate, $r_{0}$ is the baseline event rate at the mean age, ${HR}_{age}$ is the hazard ratio for age derived from the Clinical Practice Research Data Chain (CPRD) (Danese et al., 2021), Δage is the difference between the mean age of the target population and that of the cohort from which the baseline rate was obtained, RR is the rate ratio per 1.0 mmol/L LDL-C reduction, with the CTTC reporting a rate ratio (RR) of 0.78 (95% CI: 0.76–0.80) for any major vascular event, and ΔLDLc is the difference in LDL-C (mmol/L) between the target population and the cohort providing the baseline rates.

Transition probabilities were estimated based on the CTTC meta-analysis, which links LDL-C reduction to CV risk reduction (Cholesterol Treatment Trialists' Collaboration et al., 2010), an approach widely adopted in previous cost-effectiveness studies of PCSK9 inhibitors (Xie et al., 2023; Fonarow et al., 2017; Wan et al., 2025). To capture the increased risks following a prior CV event, we applied risk multipliers from the re-estimated REACH model (Table 1) (Danese et al., 2021). The LDL-C–lowering effects of PCSK9 inhibitors, in addition to standard statin therapy, were derived from a network meta-analysis comparing multiple PCSK9 inhibitor dosing regimens with placebo added to statins. All regimens showed significant LDL-C reductions (Table 1) (Liu et al., 2024). Based on CTTC findings, each 1.0 mmol/L (38.7 mg/dL) LDL-C reduction was associated with a 26% lower risk of MI, 20% lower risk of stroke, 14% lower risk of vascular death, and 24% lower risk of coronary revascularization (Cholesterol Treatment Trialists' Collaboration et al., 2010; Cholesterol Treatment Trialists’ Collaboration et al., 2015). Event rates after treatment were estimated using Equation 2:

$$r_{tx}=r_{0}\times {RR}^{\Delta LDLc}$$ (2)

where $r_{tx}$ is the post-treatment event rate, $r_{0}$ is the baseline event rate, RR is the rate ratio per 1.0 mmol/L LDL-C reduction, and ΔLDLc is the absolute LDL-C reduction (mmol/L) (Table 1).

**TABLE 1:** Key inputs in the model.

Parameter	Base value	Range	Distribution	Source
Event rates (per 100 patient-years, CTTC)				Cholesterol Treatment Trialists' Collaboration et al. (2010)
Nonfatal MI	0.9	NA	Fixed	
Nonfatal stroke	0.4	NA	Fixed	
Coronary revascularization	1.2	NA	Fixed	
Risk multipliers after prior events (HRs)				Danese et al. (2021)
Recurrent MI	1.13	(1.04, 1.22)	Lognormal	
Recurrent stroke	1.13	(0.99, 1.30)	Lognormal	
Any CV event in past year	1.50	(1.39, 1.62)	Lognormal	
Recurrent MI (≥2)	1.19	(1.05, 1.34)	Lognormal	
Recurrent stroke (≥2)	1.36	(1.03, 1.80)	Lognormal	
RR per 1 mmol/L LDL-C reduction				Cholesterol Treatment Trialists' Collaboration et al. (2010)
MI	0.74	(0.69, 0.78)	Lognormal	
Stroke	0.80	(0.73, 0.88)	Lognormal	
Coronary revascularization	0.76	(0.73, 0.80)	Lognormal	
Vascular death	0.86	(0.82, 0.90)	Lognormal	
Any death	0.90	(0.87, 0.93)	Lognormal	
Absolute LDL-C reduction (mmol/L)				Liu et al. (2024)
Alirocumab 75 mg Q2W	1.36	(1.25 1.46)	Normal	
Alirocumab 150 mg Q2W	1.47	(1.24, 1.69)	Normal	
Alirocumab 300 mg Q4W	1.53	(1.26, 1.79)	Normal	
Evolocumab 140 mg Q2W	1.79	(1.65, 1.93)	Normal	
Evolocumab 420 mg Q4W	1.59	(1.48, 1.71)	Normal	
Tafolecimab 150 mg Q2W	1.48	(1.04, 1.93)	Normal	
Tafolecimab 450 mg Q4W	1.65	(1.46, 1.85)	Normal	
Tafolecimab 600 mg Q6W	1.41	(1.10, 1.73)	Normal	
Inclisiran 300 mg Q6M	1.27	(1.11, 1.43)	Normal	
Drug costs per dose ($)				Yaozhi Database (2025)
Alirocumab (75 mg)	40.4	(32.3, 48.5)	Gamma	
Evolocumab (140 mg)	39.4	(31.5, 47.3)	Gamma	
Tafolecimab (150 mg)	39.7	(31.8, 47.7)	Gamma	
Inclisiran (300 mg)	1,387.2	(1,109.8, 1,664.7)	Gamma	
Event costs ($)				
MI (year 1)	3,275.0	(2,620.0, 3,930.0)	Gamma	National Health Commission of China (2023)
MI (subsequent)	1,893.9	(1,420.3, 2,367.3)	Gamma	Wan et al. (2025)
IS (year 1)	1,390.4	(1,112.3, 1,668.5)	Gamma	National Health Commission of China (2023)
IS (subsequent)	1,141.1	(1,099.3, 1,206.0)	Gamma	(Pan et al., 2020; Wan et al., 2025)
Revascularization	16,543.2	(13,234.6, 19,851.9)	Gamma	National Health Commission of China (2022)
CV death	2,332.7	(1,866.2, 2,799.3)	Gamma	National Health Commission of China (2023)
Utilities				
Baseline	0.964	(0.959, 0.969)	Beta	Wang et al. (2015)
MI (year 1)	0.866	(0.847, 0.886)	Beta	(Dreyer et al., 2019; Dou et al., 2022)
MI (beyond 1 year)	0.950	(0.942, 0.958)	Beta	(Dou et al., 2022; Zhang et al., 2021)
MI 2+ (year 1)	0.819	(0.793, 0.846)	Beta	Dou et al. (2022)
MI 2+ (beyond 1 year)	0.940	(0.905, 0.975)	Beta	(Dou et al., 2022; Zhang et al., 2021)
IS (year 1)	0.510	(0.470, 0.540)	Beta	Du et al. (2018)
IS (beyond 1 year)	0.750	(0.710, 0.800)	Beta	(Du et al., 2018; She et al., 2021)
IS 2+ (year 1)	0.340	(0.320, 0.360)	Beta	Wang et al. (2014)
IS 2+ (beyond 1 year)	0.420	(0.390, 0.451)	Beta	Wang et al. (2014)
Injection-site reaction (disutility)	−0.0003	(-0.002, 0)	Gamma	Fonarow et al. (2017)
Revascularization (disutility)	−0.007	(-0.014, −0.001)	Gamma	Kazi et al. (2016)

Abbreviations: CTTC, Cholesterol Treatment Trialists’ Collaboration; CV, cardiovascular; HR, hazard ratio; IS, ischemic stroke; LDL-C, low-density lipoprotein cholesterol; MI, myocardial infarction; NA, not applicable; Q2W, every 2 weeks; Q4W, every 4 weeks; Q6W, every 6 weeks; Q6M, every 6 months; RR, rate ratio. Note: distribution marked as ‘fixed’ were reported without confidence intervals in the original sources and were not varied in sensitivity analyses.

Finally, event-specific annual rates were converted to transition probabilities using the standard exponential formula (Equation 3) (Gidwani and Russell, 2020):

$$P=1-\exp\left(-rt\right)$$ (3)

where *P* represents the annual transition probability, r is the annual event rate, and t is the cycle length (1 year).

All calculated transition probabilities between health states are provided in Supplementary Table S3.

### 2.4 Mortality

Age-specific all-cause mortality rates were derived from the 2023 China Health Statistics Yearbook (National Health Commission of China, 2023), representing baseline mortality in the general Chinese population (Supplementary Table S4). CV mortality was extracted from the same source, and non-CV mortality was calculated by subtracting CV mortality from all-cause mortality. To reflect the increased risk of death after a cardiovascular event, patients with an MI or IS in the previous year were assigned a relative risk of CV death of 1.31 (95% CI: 1.15–1.49) (Wilson et al., 2012). All mortality rates were then converted into transition probabilities using Equation 3.

### 2.5 Costs and utilities

We conducted the analysis from the perspective of the Chinese healthcare system, considering only direct medical costs. These included hospitalization expenses (e.g., surgical, examination, and nursing fees) and medication costs, while direct non-medical costs, such as accommodation and transportation, were excluded. Hospitalization costs were obtained from the 2023 China Health Statistics Yearbook (National Health Commission of China, 2023). Long-term post-discharge treatment costs for myocardial infarction were derived from basic health insurance data (Wang et al., 2018; Xiong et al., 2018). Stroke subsequent annual costs were estimated from the expenses of secondary preventive treatments (Pan et al., 2020; Wan et al., 2025). Due to data limitations, we assumed the cost of CV deaths equaled the average hospitalization costs for acute MI and stroke in Chinese public hospitals (National Health Commission of China, 2023), whereas non-CV deaths were considered cost-free and independent of treatment regimens, consistent with previous studies (Xie et al., 2023; Fonarow et al., 2017). As in previous models (Wan et al., 2023; Xie et al., 2023), revascularization (RV) was considered a procedure incurring treatment costs but was not modeled as a separate health state. Outpatient follow-up and monitoring costs (e.g., lipid panels, physician visits) were not included, as these are similar between treatment groups and comprehensive national data are limited.

We sourced drug acquisition costs from publicly available Chinese databases. Unit prices for PCSK9 inhibitors were retrieved from the China Yaozhi website, while statin prices were based on the highest bid prices from the Chinese centralized procurement platform. Using the median daily cost of atorvastatin and rosuvastatin, we calculated an annual statin cost of USD 846.1 per patient (Supplementary Table S5). All costs were converted to 2024 U.S. dollars (USD) using an exchange rate of 1 USD = 7.20 Chinese Yuan (CNY) and adjusted for inflation using the Consumer Price Index (CPI) from the World Bank (https://data.worldbank.org/indicator/PA.NUS.FCRF) (Turner et al., 2019).

We derived health state utility values from published studies of Chinese populations, primarily measured using the Chinese version of the European Quality of Life 5-Dimensional Three-Level Scale (EQ-5D-3L) and its associated scoring algorithm. We incorporated temporary disutilities associated with RV or injection-related discomfort, based on previous literature (Wan et al., 2023; Xie et al., 2023; Xi et al., 2023).

Following the 2020 China Guidelines for Pharmacoeconomic Evaluations (Liu, 2020), we applied 5% annual discount rate to both costs and health outcomes and applied half-cycle corrections to all health states and costs, accounting for transitions occurring, on average, midway through each model cycle.

### 2.6 Outcomes

The primary outcome was the incremental cost-utility ratio (ICUR), defined as the additional cost per quality-adjusted life year (QALY) gained with PCSK9 inhibitors versus statin therapy alone. Health outcomes were expressed as QALYs, with incremental QALYs (ΔQALYs) and costs (ΔCosts) calculated as differences between treatment arms. ICUR was computed as ΔCosts/ΔQALYs. ICURs were calculated only among non-dominated strategies. A strategy was deemed dominated if it was more costly and less effective than an alternative, or subject to extended dominance if its ICUR exceeded that of a more effective option.

The willingness-to-pay (WTP) threshold was set at 1–3 times the 2024 national gross domestic product (GDP) *per capita* ($13,291.7–$39,875.0 per QALY) (Liu, 2020; Griffiths et al., 2015), which is established by the World Health Organization for cost-effectiveness and China Guidelines for Pharmacoeconomic Evaluations (Liu, 2020; World Health Organization, 2025). Cost-effectiveness was categorized as: (1) ICUR <1 × GDP/QALY, highly cost-effective; (2) ICUR 1–3 × GDP/QALY, cost-effective; and (3) ICUR >3 × GDP/QALY, not cost-effective.

To further explore value-based pricing, a price threshold analysis was performed to estimate the maximum price at which PCSK9 inhibitors would be considered cost-effective under the specified WTP threshold.

### 2.7 Sensitivity analyses

Deterministic and probabilistic sensitivity analyses were performed to evaluate the robustness of the model results. In the one-way sensitivity analysis, key parameters—including treatment costs, utility values, efficacy estimates, and RRs for cardiovascular events—were varied individually within their 95% confidence intervals (CIs), or by ±20% of base-case values when CIs were unavailable. The annual discount rate was varied between 0% and 8%. Results were presented as tornado diagrams.

For the probabilistic sensitivity analysis (PSA), 1,000 Monte Carlo simulations were performed to account for joint parameter uncertainty. Standard probability distributions were assigned: gamma for costs, lognormal for RRs, normal for mean LDL-C reduction, and beta for transition probabilities and utility values (Table 1). PSA results were illustrated using scatter plots and cost-effectiveness acceptability curves (CEACs).

Scenario analyses were also conducted to assess the impact of treatment duration, with alternative time horizons ranging from 5 to 30 years.

### 2.8 Model validation

The model was adapted from previously published and validated economic evaluation frameworks for lipid-lowering therapies. Therefore, it underwent validation for face and internal validity, but not independent external validation against specific trial data.

Face validity was established through consultations with clinical pharmacists and health economists, who reviewed the model structure, assumptions, input parameters, and outputs to ensure clinical relevance and logical consistency. Internal validity was verified by comprehensive checks of parameter sources, formula integrity, and computational logic. Independent cross-checking by another researcher confirmed the accuracy of the model’s implementation and results.

## 3 Results

### 3.1 Base-case analysis

In the base case, using a network meta-analysis of the hypercholesterolemia population with a mean baseline LDL-C of 3.13 mmol/L, statin therapy alone was the least costly option, with a mean lifetime cost of $13,706.18 and effectiveness of 12.32 QALYs. Among active comparators, evolocumab 140 mg every 2 weeks (Q2W) was the only non-dominated option. It yields a mean lifetime cost of $24,815.45 and effectiveness of 12.74 QALYs, corresponding to incremental costs of $11,109.27 and a QALY gain of 0.42 versus statins alone. The resulting ICUR was $26,217.47 per QALY gained, which is well within the WTP threshold of 1–3× GDP per QALY ($39,875/QALY), indicating cost-effectiveness (Table 2). All other regimens, including various doses of alirocumab (75–300 mg), tafolecimab (150–600 mg), evolocumab 420 mg every 4 weeks (Q4W), and inclisiran 300 mg every 6 months (Q6M), were absolutely dominated, showing lower QALY gains at higher costs compared with evolocumab 140 mg Q2W.

**TABLE 2:** Base-case cost-utility results.

Treatment strategy	Total cost ($)	QALYs	ΔCosts ($)	ΔQALYs	ICUR vs. statins ($/QALY)	^a^ ICUR vs. previous non-dominated strategy ($/QALY)
Statins alone	13,706.18	12.32	NA	NA	NA	NA
Evolocumab 140 mg Q2W	24,815.45	12.74	11,109.27	0.42	26,217.47	26,217.47
Alirocumab 75 mg Q2W	24,946.32	12.64	11,240.14	0.33	34,279.73	Dominated
Tafolecimab 150 mg Q2W	25,767.16	12.67	12,060.98	0.35	34,002.10	Dominated
Tafolecimab 600 mg Q6W	29,879.66	12.66	16,173.49	0.34	47,716.08	Dominated
Evolocumab 420 mg Q4W	30,934.51	12.70	17,228.34	0.38	45,392.54	Dominated
Tafolecimab 450 mg Q4W	31,117.00	12.71	17,410.82	0.39	44,317.10	Dominated
Alirocumab 150 mg Q2W	37,624.59	12.67	23,918.41	0.35	67,814.11	Dominated
Alirocumab 300 mg Q4W	37,679.37	12.68	23,973.20	0.37	65,473.09	Dominated
Inclisiran 300 mg Q6M	48,693.34	12.62	34,987.16	0.31	113,800.14	Dominated

^a^ ICUR, compares each strategy with the previous less costly, excluding dominated strategies. Abbreviations: ICUR, incremental cost-utility ratio; NA, not applicable; Q2W, every 2 weeks; Q4W, every 4 weeks; Q6W, every 6 weeks; Q6M, every 6 months; QALY, quality-adjusted life years.

However, when compared individually to statins alone, the ICURs for alirocumab 75 mg Q2W, and tafolecimab 150 mg Q2W were all below 3× GDP/QALY ($39,875/QALY), indicating cost-effectiveness. Notably, inclisiran showed the least favorable profile, with a lifetime cost of $48,693.34 and 12.62 QALYs, resulting in an ICUR of $113,800.14 per QALY gained versus statins alone.

### 3.2 Sensitivity analyses

**FIGURE 2:**
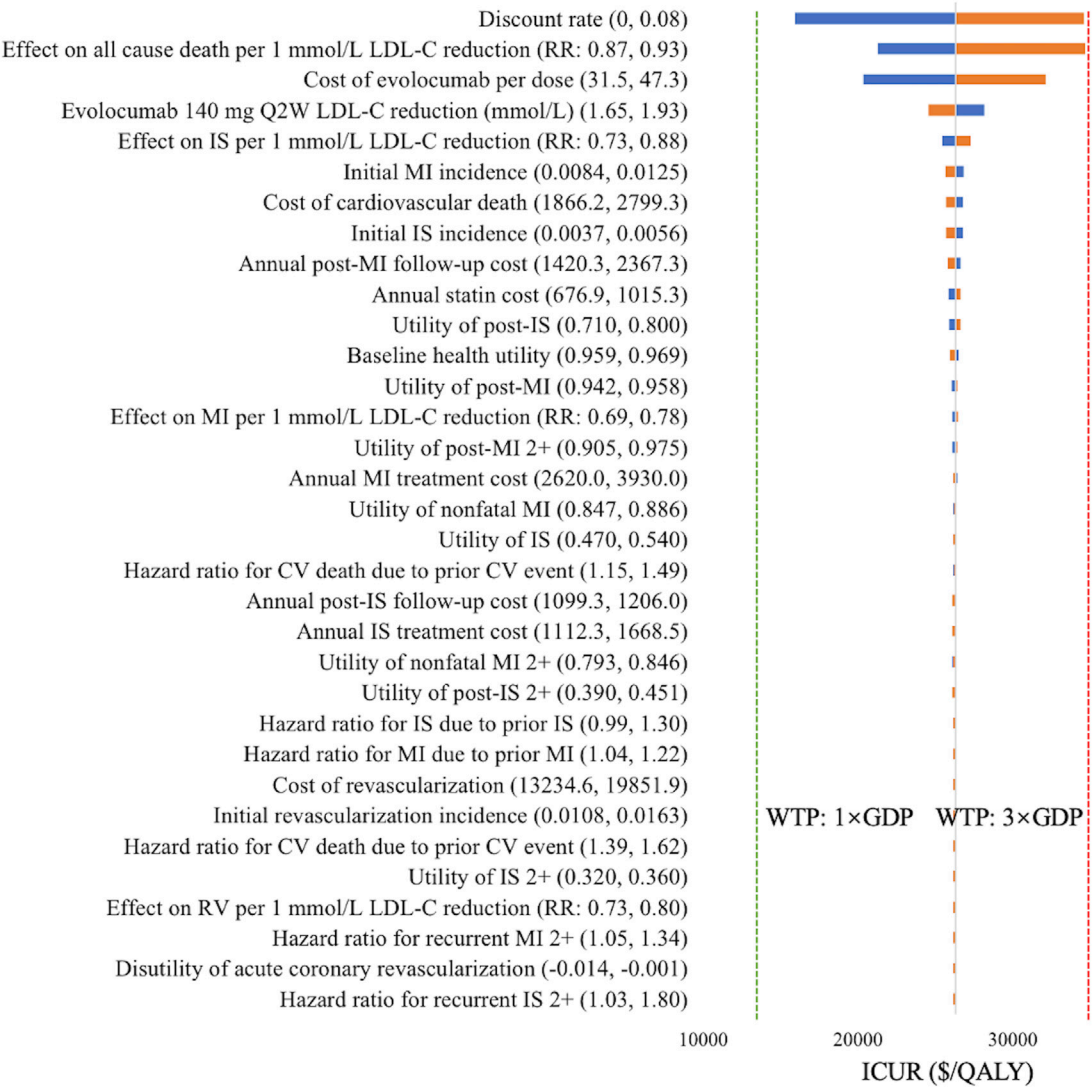
Tornado diagram of evolocumab 140 mg Q2W versus statin therapy alone. Blue bars indicate ICURs at the lower parameter values, and yellow bars indicate ICURs at the upper parameter values. The green dotted line denotes the WTP threshold of one time GDP *per capita* ($13,291.7/QALY), and the red dotted line denotes the WTP threshold of three times GDP *per capita* ($39,875/QALY). Abbreviations: CV, cardiovascular; GDP, gross domestic product; ICUR, incremental cost-utility ratio; IS, ischemic stroke; LDL-C, low-density lipoprotein cholesterol; MI, myocardial infarction; Q2W, every 2 weeks; QALY, quality-adjusted life years; RR, rate ratio; RV, revascularization; WTP, willingness-to-pay.

The one-way sensitivity analysis confirmed the robustness of base-case results when individual parameters were varied. As shown in Figure 2, the parameters with the greatest impact on the ICUR of evolocumab 140 mg Q2W versus statins alone were discount rate (ICUR range: $15,903.46–$34,573.62/QALY), the effect of LDL-C reduction on all-cause death ($21,234.78–$34,696.08/QALY), and the cost of evolocumab per dose ($20,322.14–$32,112.78/QALY). Despite these variations, all ICURs remained below the WTP threshold of 3× GDP per QALY ($39,875/QALY).

**FIGURE 3:**
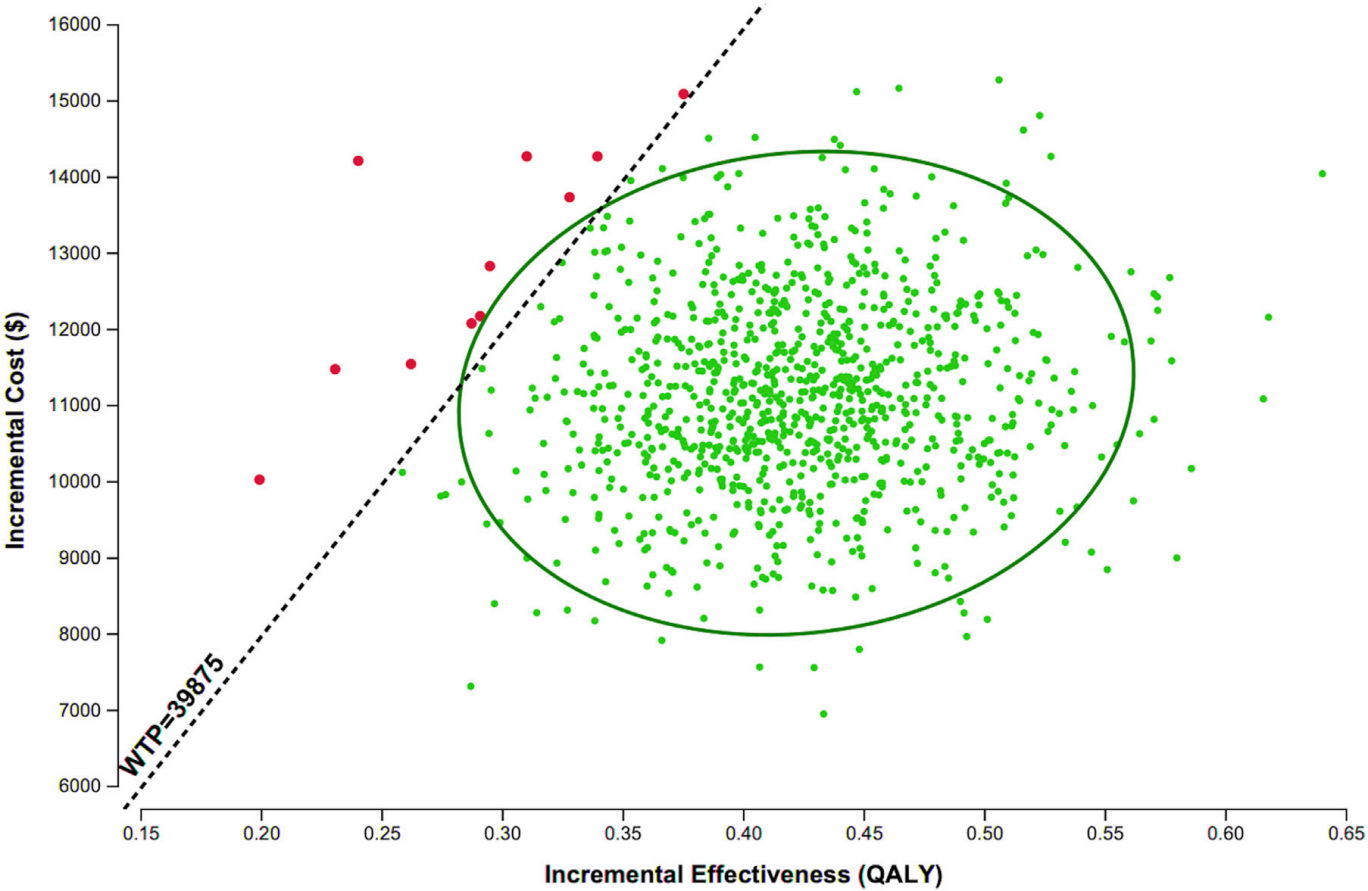
Scatter plot of evolocumab 140 mg Q2W versus statin therapy alone. Each point represents the corresponding incremental costs and QALYs. Abbreviations: QALY, quality-adjusted life years; WTP, willingness-to-pay.

For the PSA, we performed 1,000 Monte-Carlo simulations based on the specified ranges and distributions of model parameters. As shown in Figure 3, the majority of simulations fell below the WTP threshold of 3× GDP per QALY ($39,875/QALY), indicating that adding evolocumab Q2W to statins has a 98.9% probability of being cost-effective.

**FIGURE 4:**
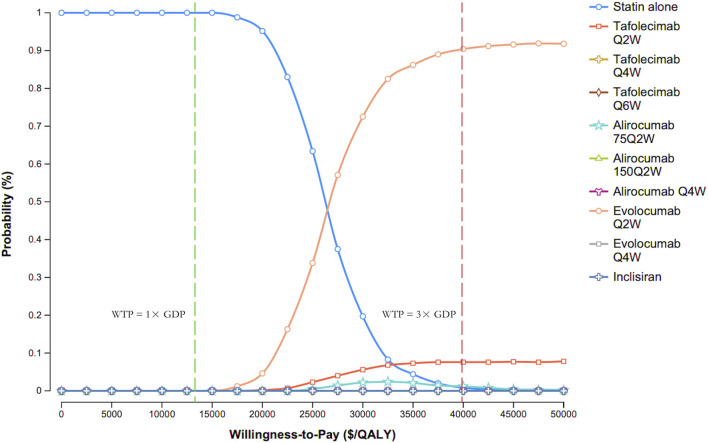
Cost-effectiveness acceptability curves. Abbreviations: GDP, gross domestic product; Q2W, every 2 weeks; Q4W, every 4 weeks; Q6W, every 6 weeks; WTP, willingness-to-pay.

Figure 4 presents the CEAC for various PCSK9 inhibitor regimens versus statin therapy alone. At the WTP threshold of 3× GDP per QALY, evolocumab Q2W had a 90.3% probability of being cost-effective, compared with 7.6% for tafolecimab Q2W, 1.3% for alirocumab 75 mg Q2W, 0.8% for statin therapy alone, and 0% for all other treatments.

### 3.3 Scenario analysis

A scenario analysis varying the time horizon from 5 to 30 years showed progressively lower ICURs for evolocumab versus statin therapy alone: $220,505.85/QALY (5 years) to $26,217.47/QALY (30 years) (Table 3). At the WTP threshold of $39,875/QALY, evolocumab 140 mg Q2W was not cost-effective for horizons ≤20 years but became cost-effective at 25 and 30 years, indicating that longer horizons significantly improve its economic value under current pricing. Similar trends were observed for other PCSK9 inhibitor regimens (Supplementary Table S6).

**TABLE 3:** Scenario analysis of evolocumab 140 mg Q2W vs. statins across different treatment durations.

Time horizon (years)	Statins cost ($)	Statins QALY	Evolocumab Q2W cost ($)	Evolocumab Q2W QALY	ΔCost($)	ΔQALY	ICUR ($/QALY)
5	3,911.29	4.18	8,200.20	4.20	4,288.91	0.02	220,505.85
10	7,080.83	7.28	14,330.66	7.35	7,249.83	0.07	107,429.44
15	9,570.87	9.50	18,786.11	9.64	9,215.24	0.14	66,446.92
20	11,454.75	11.00	21,874.16	11.23	10,419.42	0.23	45,644.45
25	12,809.73	11.91	23,836.69	12.24	11,026.96	0.33	33,610.33
30	13,706.18	12.32	24,815.45	12.74	11,109.27	0.42	26,217.47

Abbreviations: Q2W, every 2 weeks; QALY, quality-adjusted life years; ICUR, incremental cost-utility ratio.

### 3.4 Price threshold analysis

At current prices, evolocumab 140 mg Q2W, alirocumab 75 mg Q2W, and tafolecimab 150 mg Q2W were already cost-effective compared with statin therapy alone, requiring no price reduction. In contrast, regimens such as tafolecimab 600 mg every 6 weeks (Q6W), evolocumab 420 mg Q4W, and alirocumab 150 mg Q2W would need moderate price reductions of 9%–39% to reach cost-effectiveness. Inclisiran 300 mg Q6M, which exhibited the highest ICUR in the base-case analysis, would require a substantial price reduction of approximately 63% to meet the 3× GDP threshold (Table 4).

**TABLE 4:** Price-threshold results for cost-utility at a threshold of 3× GDP per QALY ($39,875/QALY).

Treatment strategy	Base case ICUR ($/QALY)	Maximum cost per dose at 3× GDP threshold ($)	Required price change at 3× GDP threshold (%)
Evolocumab 140 mg Q2W	26,217.47	No reduction needed	NA
Alirocumab 75 mg Q2W	34,279.73	No reduction needed	NA
Tafolecimab 150 mg Q2W	34,002.10	No reduction needed	NA
Tafolecimab 600 mg Q6W	47,716.08	33.3	−16.1
Evolocumab 420 mg Q4W	45,392.54	35.0	−11.3
Tafolecimab 450 mg Q4W	44,317.10	36.0	−9.3
Alirocumab 150 mg Q2W	67,814.11	24.6	−39.0
Alirocumab 300 mg Q4W	65,473.09	25.4	−37.0
Inclisiran 300 mg Q6M	113,800.14	508.8	−63.3

Abbreviations: GDP, gross domestic product; ICUR, incremental cost-utility ratio; NA, not applicable; Q2W, every 2 weeks; Q4W, every 4 weeks; Q6W, every 6 weeks; Q6M, every 6 months; QALY, quality-adjusted life years.

## 4 Discussion

This study evaluated the cost-utility of PCSK9 inhibitors (evolocumab, alirocumab, tafolecimab, and inclisiran) as adjuncts to statin therapy in Chinese patients with hypercholesterolemia or at high cardiovascular risk, using a Markov cohort multistate-transition model informed by LDL-C reductions from a network meta-analysis. To our knowledge, this is the first comprehensive pharmacoeconomic comparison of all PCSK9 inhibitors currently marketed in China from the perspective of the Chinese healthcare system, evaluating primary prevention in hypercholesterolemia.

In the base-case analyses, evolocumab 140 mg Q2W was the most cost-effective regimen, with an ICUR of $26,217.47/QALY versus statin therapy alone, well below the Chinese WTP threshold ($39,875/QALY). While evolocumab dominates other regimens, alirocumab 75 mg Q2W and tafolecimab 150 mg Q2W were also cost-effective compared with statin therapy alone under current negotiated prices.

Previous pharmacoeconomic evaluations of PCSK9 inhibitors in China, including studies of evolocumab (Wan et al., 2023; Xie et al., 2023; Xi et al., 2023; Liang et al., 2021b), alirocumab (Liang et al., 2021a), tafolecimab (Wan et al., 2025), and inclisiran (Zhou et al., 2024), predominantly adopted the healthcare system perspective, with one also incorporating the patient perspective (Wan et al., 2023). All employed Markov cohort state-transition models, varying in health states and model structure.

Before evolocumab’s inclusion in the National Drug List (2021) at CNY 1,298 ($180.3) per dose, results were mixed: it was not cost-effective in post-MI patients (Liang et al., 2021b) but cost-effective in acute coronary syndrome, particularly for very high-risk ASCVD patients (Xi et al., 2023), likely due to methodological differences. After national price negotiations reduced the price to CNY 283.8 ($39.4) per dose, evolocumab plus statins became cost-effective (Xie et al., 2023), consistent with our findings.

Similarly, alirocumab was initially not cost-effective at CNY 1,982 ($275.3)/dose, requiring an 88% price reduction to meet the WTP threshold (Liang et al., 2021a). At its current reimbursed price of $40.4 per dose, alirocumab 75 mg Q2W is cost-effective versus statins alone (ICUR $34,279.73/QALY).

Tafolecimab, a domestic PCSK9 inhibitor with flexible dosing options, was approved by the National Medical Products Administration (NMPA) in 2023 and added to the national medical insurance list in 2024. Although less cost-effective than evolocumab, tafolecimab 150 mg Q2W remained cost-effective versus statins alone at its current price of $39.7 per dose, similar to a prior threshold analysis (Wan et al., 2025).

Inclisiran was the least favorable option, with the highest lifetime costs and lowest QALYs among the therapies assessed. Despite price reductions to CNY 9,988 ($1,387) per dose, its ICUR remains above the WTP threshold, consistent with prior studies estimating it would only be cost-effective with over 88% price reduction (Zhou et al., 2024). Similar international findings highlight the high price sensitivity of inclisiran (Kam et al., 2020; Galactionova et al., 2022; Desai et al., 2022).

All PCSK9 inhibitors improved QALYs relative to statins alone (Jena et al., 2016; Kazi et al., 2016), supporting broader population health benefits. Reductions in cardiovascular events, hospitalizations, and revascularizations contributed to a favorable cost-utility. Under current pricing in China, evolocumab is the most economically attractive option, with alirocumab and tafolecimab as viable alternatives, while further price adjustments would be needed to justify inclisiran in routine practice.

Because most trials have relatively short follow-up for hard outcomes, our model projected lifetime cardiovascular benefits by linking LDL-C reductions to event risk, supported by CTTC meta-analyses (Cholesterol Treatment Trialists' Collaboration et al., 2010; Cholesterol Treatment Trialists’ Collaboration et al., 2015; Cholesterol Treatment Trialists’ Collaboration, 2019). Mendelian randomization studies reinforce this approach, showing comparable reductions in coronary mortality for lifelong LDL-C lowering via 3-hydroxy-3-methylglutaryl-coenzyme A (HMG-CoA) reductase and PCSK9 pathways (Ference et al., 2016). Using this framework, we translated each regimen’s LDL-C reduction into proportional changes in MACE to estimate QALYs and costs. This approach aligns with prior economic evaluations of PCSK9 inhibitors that also projected outcomes from LDL-C reductions (Fonarow et al., 2017; Xie et al., 2023; Kazi et al., 2016; Gandra et al., 2016). LDL-C anchored models, by relying on a validated dose-response relationship, provide more stable estimates across settings and allow indirect comparisons and long-term extrapolation (Korman and Wisloff, 2018). In contrast, some models rely on trial-reported hazard ratios or country-specific risk equations (Villa et al., 2020), but their outcomes are more sensitive to trial populations, event definitions, and assumptions about cardiovascular mortality effects, thereby limiting generalizability (Fonarow et al., 2017).

Scenario analyses indicated that extending the time horizon substantially improved the cost-utility of evolocumab. The ICUR declined markedly with longer treatment durations, from $220,505.85/QALY at 5 years to $45,644.45/QALY at 20 years (approximately the average life expectancy in China) and further to $26,217.47/QALY at 30 years, covering nearly the entire population lifespan. This trend is consistent with the economic principles of chronic disease management, where the benefits of effective therapies accumulate over time. By delaying disease progression and reducing the incidence of major adverse cardiovascular events (e.g., myocardial infarction and stroke), long-term treatment can generate considerable health gains and offset significant costs (Mohiuddin et al., 2024). These events are not only associated with high direct medical expenditures but also with substantial losses in health-related quality of life. Consequently, sustained therapy reduces both the clinical and economic burden of disease, as reflected in the growing QALY gains and the declining ICURs observed in our analysis. Furthermore, the discount rate applied in the model influenced long-term cost-effectiveness estimates. When early clinical benefits persist and downstream costs plateau, a long-term perspective becomes essential to fully capture the economic value of high-cost preventive therapies (Hultkrantz, 2021).

This study has several limitations that should be acknowledged. First, the model’s clinical efficacy inputs were derived from a network meta-analysis with relatively short follow-up, while long-term benefits were extrapolated over a lifetime. However, if LDL-C–lowering effects or cardiovascular risk reductions attenuate over time, the actual cost-effectiveness of PCSK9 inhibitors could be over- or underestimated. Moreover, in the absence of head-to-head trials comparing different PCSK9 inhibitors and dosing regimens, efficacy estimates relied on indirect evidence, introducing additional uncertainty. Second, the relationship between LDL-C reduction and CV risk reduction was based on the CTTC meta-analysis. If the real-world CV risk reduction associated with PCSK9 inhibitors differs from this estimate, the cost-effectiveness results may be biased. Third, due to the lack of real-world data in Chinese patients, baseline CV event rates and many transition probabilities were drawn from international literature, potentially limiting the generalizability of the findings to the Chinese healthcare setting. Fourth, this analysis was conducted from the Chinese healthcare system perspective and included only direct medical costs. Exclusion of indirect costs and broader societal benefits, such as productivity gains from improved long-term outcomes, may underestimate the full economic value of PCSK9 inhibitors. Finally, the model assumed full adherence throughout the time horizon, whereas real-world treatment persistence is typically lower. Although some studies report variable adherence and persistence of PCSK9 inhibitors (Svensson et al., 2024; Gargiulo et al., 2023), their relatively short follow-up does not capture long-term patterns. Consequently, persistence and adherence were not included in the base-case analysis, which may have led to overestimated QALY gains and cost-utility.

## 5 Conclusion

This study provides the first comprehensive pharmacoeconomic evaluation of all currently available PCSK9 inhibitors in China. Using a Markov multistate-transition model, we found that evolocumab 140 mg Q2W emerged as the most cost-effective regimen for patients with hypercholesterolemia from the Chinese healthcare perspective, with ICUR well below the willingness-to-pay threshold. Alirocumab 75 mg Q2W and tafolecimab 150 mg Q2W were also identified as cost-effective alternatives. These results offer valuable evidence to guide clinical decision-making and optimize healthcare resource allocation.

## Data Availability

The original contributions presented in the study are included in the article/Supplementary Material, further inquiries can be directed to the corresponding authors.
