# Supplementary material for: Cost-utility analysis of PCSK9 inhibitors for hypercholesterolemia: a Chinese healthcare perspective
Source: Front Pharmacol. 2025 Nov 19;16:1708701. doi: 10.3389/fphar.2025.1708701 (PMC12672326; doi:10.3389/fphar.2025.1708701)
Supplement: Supplementary file 1 [file Supplementaryfile1.docx]

Supplementary Material

# **Supplementary Tables**

Table S1 Consolidated Health Economic Evaluation Reporting Standards 2022 (CHEERS 2022) [1]

| **Topic** | **No.** | **Item** | **Location where item is reported** |
| --- | --- | --- | --- |
| **Title** |  |  |  |
|  | 1 | Identify the study as an economic evaluation and specify the interventions being compared. | Title page |
| **Abstract** |  |  |  |
|  | 2 | Provide a structured summary that highlights context, key methods, results, and alternative analyses. | Abstract |
| **Introduction** |  |  |  |
| Background and objectives | 3 | Give the context for the study, the study question, and its practical relevance for decision making in policy or practice. | Introduction |
| **Methods** |  |  |  |
| Health economic analysis plan | 4 | Indicate whether a health economic analysis plan was developed and where available. | Available from the corresponding author upon request |
| Study population | 5 | Describe characteristics of the study population (such as age range, demographics, socioeconomic, or clinical characteristics). | Section "Methods", subsection "Patient population"; Supplementary Table S2 |
| Setting and location | 6 | Provide relevant contextual information that may influence findings. | Section "Methods", subsection "Model structure" |
| Comparators | 7 | Describe the interventions or strategies being compared and why chosen. | Section "Methods", subsection "Patient population" |
| Perspective | 8 | State the perspective(s) adopted by the study and why chosen. | Section "Methods", subsection "Costs and utilities" |
| Time horizon | 9 | State the time horizon for the study and why appropriate. | Section "Methods", subsection "Model structure" |
| Discount rate | 10 | Report the discount rate(s) and reason chosen. | Section "Methods", subsection "Costs and utilities" |
| Selection of outcomes | 11 | Describe what outcomes were used as the measure(s) of benefit(s) and harm(s). | Section "Methods", subsection "Outcomes" |
| Measurement of outcomes | 12 | Describe how outcomes used to capture benefit(s) and harm(s) were measured. | Section "Methods", subsections "Costs and utilities" and "Outcomes" |
| Valuation of outcomes | 13 | Describe the population and methods used to measure and value outcomes. | Not Applicable |
| Measurement and valuation of resources and costs | 14 | Describe how costs were valued. | Section "Methods", subsection "Costs and utilities" |
| Currency, price date, and conversion | 15 | Report the dates of the estimated resource quantities and unit costs, plus the currency and year of conversion. | Section "Methods", subsection "Costs and utilities" |
| Rationale and description of model | 16 | If modelling is used, describe in detail and why used. Report if the model is publicly available and where it can be accessed. | Section "Methods", subsection "Model structure" |
| Analytics and assumptions | 17 | Describe any methods for analyzing or statistically transforming data, any extrapolation methods, and approaches for validating any model used. | Subsections "Clinical event probabilities and intervention effects" and "Mortality" |
| Characterizing heterogeneity | 18 | Describe any methods used for estimating how the results of the study vary for subgroups. | Subsections "Patient population", Supplementary Table S2 |
| Characterizing distributional effects | 19 | Describe how impacts are distributed across different individuals or adjustments made to reflect priority populations. | Not applicable |
| Characterizing uncertainty | 20 | Describe methods to characterize any sources of uncertainty in the analysis. | Subsection "Sensitivity analyses" |
| Approach to engagement with patients and others affected by the study | 21 | Describe any approaches to engage patients or service recipients, the general public, communities, or stakeholders (such as clinicians or payers) in the design of the study. | Not applicable |
| **Results** |  |  |  |
| Study parameters | 22 | Report all analytic inputs (such as values, ranges, references) including uncertainty or distributional assumptions. | Table 1 |
| Summary of main results | 23 | Report the mean values for the main categories of costs and outcomes of interest and summarise them in the most appropriate overall measure. | Subsection "Base-case analysis" |
| Effect of uncertainty | 24 | Describe how uncertainty about analytic judgments, inputs, or projections affect findings. Report the effect of choice of discount rate and time horizon, if applicable. | Subsection "Sensitivity analyses" and "Scenario Analyses" |
| Effect of engagement with patients and others affected by the study | 25 | Report on any difference patient/service recipient, general public, community, or stakeholder involvement made to the approach or findings of the study | Not applicable |
| **Discussion** |  |  |  |
| Study findings, limitations, generalizability, and current knowledge | 26 | Report key findings, limitations, ethical or equity considerations not captured, and how these could affect patients, policy, or practice. | Section Discussion |
| **Other relevant information** |  |  |  |
| Source of funding | 27 | Describe how the study was funded and any role of the funder in the identification, design, conduct, and reporting of the analysis | Subsection "Funding" |
| Conflicts of interest | 28 | Report authors conflicts of interest according to journal or International Committee of Medical Journal Editors requirements. | Subsection "Conflicts of Interest" |

Table S2 Baseline characteristics of the patient population

| Characteristic | Value |
| --- | --- |
| Age, years | Mean: 60.6 |
|  | Median (range): 60.8 (49.0–66.1) |
| Female Sex, % | 39.2% |
| Comorbidities, % |  |
| Diabetes Mellitus | 37.7% |
| Hypertension | 72.6% |
| Lipid Profile |  |
| Baseline LDL-C, mg/dL | 121.0 (92.6–198.7) |
| Baseline LDL-C, mmol/L | 3.13 (2.39–5.14) |

Abbreviations: LDL-C, low-density lipoprotein cholesterol.

Table S3 Transition probabilities between health states at baseline and with PCSK9 inhibitor treatment

| Event | Placebo | Alirocumab 75 mg Q2W | Alirocumab 150 mg Q2W | Alirocumab 300 mg Q4W | Evolocumab 140 mg Q2W | Evolocumab 420 mg Q4W | Tafolecimab 150 mg Q2W | Tafolecimab 450 mg Q4W | Tafolecimab 600 mg Q6W | Inclisiran 300 mg Q6M |
| --- | --- | --- | --- | --- | --- | --- | --- | --- | --- | --- |
| MI | 0.0104 | 0.0069 | 0.0067 | 0.0066 | 0.0061 | 0.0065 | 0.0067 | 0.0063 | 0.0068 | 0.0071 |
| Stroke | 0.0046 | 0.0034 | 0.0033 | 0.0033 | 0.0031 | 0.0033 | 0.0033 | 0.0032 | 0.0034 | 0.0035 |
| Revascularization | 0.0138 | 0.0095 | 0.0093 | 0.0091 | 0.0085 | 0.0090 | 0.0092 | 0.0088 | 0.0094 | 0.0098 |
| Recurrent MI | 0.0117 | 0.0078 | 0.0076 | 0.0074 | 0.0069 | 0.0073 | 0.0075 | 0.0072 | 0.0077 | 0.0080 |
| Recurrent stroke | 0.0052 | 0.0039 | 0.0038 | 0.0037 | 0.0035 | 0.0037 | 0.0038 | 0.0036 | 0.0038 | 0.0039 |
| Stroke following MI | 0.0069 | 0.0051 | 0.0050 | 0.0049 | 0.0047 | 0.0049 | 0.0050 | 0.0048 | 0.0051 | 0.0052 |
| MI following stroke | 0.0155 | 0.0104 | 0.0100 | 0.0098 | 0.0091 | 0.0097 | 0.0100 | 0.0095 | 0.0102 | 0.0106 |
| Recurrent MI (≥2) | 0.0124 | 0.0082 | 0.0080 | 0.0078 | 0.0072 | 0.0077 | 0.0079 | 0.0075 | 0.0081 | 0.0084 |
| Recurrent stroke (≥2) | 0.0063 | 0.0047 | 0.0045 | 0.0045 | 0.0042 | 0.0044 | 0.0045 | 0.0044 | 0.0046 | 0.0047 |

Abbreviations: MI, myocardial infarction; Q2W, every two weeks; Q4W, every four weeks; Q6W, every six weeks; Q6M, every six months

Table S4 Age-specific annual probabilities of cardiovascular and non-cardiovascular death in the general Chinese population

| Age | Death probability (%) | | |
| --- | --- | --- | --- |
|  | All-cause death | CV death | Non-CV death |
| 60-64 | 0.0072 | 0.0011 | 0.0061 |
| 65-69 | 0.0121 | 0.0019 | 0.0102 |
| 70-74 | 0.0199 | 0.0035 | 0.0164 |
| 75-79 | 0.0336 | 0.0067 | 0.0271 |
| 80-84 | 0.0581 | 0.0138 | 0.0449 |
| ≥85 | 0.1391 | 0.0428 | 0.1007 |

Abbreviations: CV, cardiovascular

Table S5 Costs of statin therapy

| Generic name | Specification (mg) | Cost per unit ($) | Dosage (mg/day) | Annual Cost ($) | | |
| --- | --- | --- | --- | --- | --- | --- |
|  |  |  |  | Min | Max | Median |
| Atorvastatin | 20 | 0.97 | 40-80 | 668.66 | 1023.52 | 846.09 |
| Rosuvastatin | 10 | 0.86 | 20 |  |  |  |

Table S6 Scenario analysis of treatment strategies versus placebo across different time horizons

| Treatment strategy | 5-year ICUR ($/QALY) | 10-year ICUR ($/QALY) | 15-year ICUR ($/QALY) | 20-year ICUR ($/QALY) | 25-year ICUR ($/QALY) | 30-year ICUR ($/QALY) |
| --- | --- | --- | --- | --- | --- | --- |
| Placebo | - | - | - | - | - | - |
| Alirocumab 75 mg Q2W | 288,098.06 | 140,750.10 | 87,183.42 | 59,873.72 | 44,033.41 | 34,279.73 |
| Alirocumab 150 mg Q2W | 542,788.13 | 266,940.11 | 166,555.73 | 115,383.77 | 85,783.10 | 67,814.11 |
| Alirocumab 300 mg Q4W | 523,946.58 | 257,607.89 | 160,714.04 | 111,346.83 | 82,797.88 | 65,473.09 |
| Evolocumab 140 mg Q2W | 220,505.85 | 107,429.44 | 66,446.92 | 45,644.45 | 33,610.33 | 26,217.47 |
| Evolocumab 420 mg Q4W | 369,498.03 | 181,134.20 | 112,679.08 | 77,835.82 | 57,679.16 | 45,392.54 |
| Tafolecimab 150 mg Q2W | 264,268.23 | 129,894.68 | 81,190.08 | 56,516.58 | 42,384.34 | 34,002.10 |
| Tafolecimab 450 mg Q4W | 360,505.30 | 176,692.76 | 109,912.61 | 75,938.01 | 56,289.35 | 44,317.10 |
| Tafolecimab 600 mg Q6W | 369,300.66 | 181,959.26 | 113,920.10 | 79,362.65 | 59,520.89 | 47,716.08 |
| Inclisiran 300 mg Q6M | 1,042,156.56 | 468,530.37 | 284,167.20 | 194,515.00 | 143,939.37 | 113,800.14 |

Abbreviations: ICUR, incremental cost-utility ratio; Q2W, every two weeks; Q4W, every four weeks; Q6W, every six weeks; Q6M, every six months; QALY, quality-adjusted life years

Reference

[1] HUSEREAU D, DRUMMOND M, AUGUSTOVSKI F, et al. Consolidated Health Economic Evaluation Reporting Standards 2022 (CHEERS 2022) Statement: Updated Reporting Guidance for Health Economic Evaluations [J]. Value Health, 2022, 25(1): 3-9.
